# Human Gut Microbiota for Diagnosis and Treatment of Depression

**DOI:** 10.3390/ijms25115782

**Published:** 2024-05-26

**Authors:** Olga V. Averina, Elena U. Poluektova, Yana A. Zorkina, Alexey S. Kovtun, Valery N. Danilenko

**Affiliations:** 1 Vavilov Institute of General Genetics, Russian Academy of Sciences (RAS), 119333 Moscow, Russia; epolu@vigg.ru (E.U.P.); zorkina.ya@serbsky.ru (Y.A.Z.); kovtunas25@gmail.com (A.S.K.); valerid@vigg.ru (V.N.D.); 2 V. Serbsky National Medical Research Centre of Psychiatry and Narcology, Kropotkinsky per. 23, 119034 Moscow, Russia; 3 Mental-Health Clinic No. 1 Named after N.A. Alekseev, Zagorodnoe Highway 2, 115191 Moscow, Russia

**Keywords:** depression, gut microbiota, biomarkers, machine learning, psychobiotics, new generation probiotic

## Abstract

Nowadays, depressive disorder is spreading rapidly all over the world. Therefore, attention to the studies of the pathogenesis of the disease in order to find novel ways of early diagnosis and treatment is increasing among the scientific and medical communities. Special attention is drawn to a biomarker and therapeutic strategy through the microbiota–gut–brain axis. It is known that the symbiotic interactions between the gut microbes and the host can affect mental health. The review analyzes the mechanisms and ways of action of the gut microbiota on the pathophysiology of depression. The possibility of using knowledge about the taxonomic composition and metabolic profile of the microbiota of patients with depression to select gene compositions (metagenomic signature) as biomarkers of the disease is evaluated. The use of in silico technologies (machine learning) for the diagnosis of depression based on the biomarkers of the gut microbiota is given. Alternative approaches to the treatment of depression are being considered by balancing the microbial composition through dietary modifications and the use of additives, namely probiotics, postbiotics (including vesicles) and prebiotics as psychobiotics, and fecal transplantation. The bacterium *Faecalibacterium prausnitzii* is under consideration as a promising new-generation probiotic and auxiliary diagnostic biomarker of depression. The analysis conducted in this review may be useful for clinical practice and pharmacology.

## 1. Introduction

Major depressive disorder (MDD) is a common mental illness that affects more than 350 million people worldwide. The number of undiagnosed people suffering from subclinical depressive symptoms is estimated to be even higher [1]. The number of reported cases of depression and anxiety increased by more than 25% worldwide during the COVID-19 pandemic [2]. During the period of heightened social tension in the world, which led to great economic and social losses, the frequency of depression outbreaks has also intensified.

About 20–60% of patients with psychiatric disorders are resistant to treatment, which increases healthcare burden and costs by up to 10-fold compared with patients in general [3]. Therefore, attention to the studies of the pathogenesis of the disease in order to find novel ways of early diagnosis and treatment is increasing among the scientific and medical communities.

The typical symptoms of MDD include depressed mood and/or loss of interest in life for at least two weeks. The disorder is accompanied by weight changes, agitation or psychomotor retardation, fatigue, insomnia or hypersomnia, feelings of worthlessness or guilt and suicide attempts. MDD is associated with neurodegenerative diseases, dementia, an increased risk of developing cardiovascular diseases and metabolic diseases, such as type 2 diabetes mellitus [4]. Depressive symptoms are observed in oncology, autoimmune diseases and systemic infections accompanied by chronic inflammation [5]. Today it is known that the mechanisms of pathogenesis of the disease are multifactorial and are determined by polymodal changes in the endocrine, immune, metabolic and gastrointestinal systems and the central nervous system (CNS). They also include dysfunctions of the hypothalamic–pituitary–adrenal (HPA) axis [6], immune–inflammatory and oxidative pathways [7], altered vagus nerve tone [8] and an imbalance between excitatory and inhibitory signaling [9]. Factors that can lead to the development of depression are chronic and acute stress [10], environmental factors malnutrition [11], metabolic disorders [12], hormone levels [13], depletion of monoamines [14] and also intracellular mechanisms such as mitochondrial dysfunction [15] and epigenetics [16].

The gut microbiota (GM) is currently considered as an important mechanism of the pathogenesis of depression. It has been shown that animals acquired a depressive phenotype after transplantation of intestinal flora (FMT) from patients with MDD [17]. Numerous animal and clinical studies indicate a bidirectional connection between GM and the brain through metabolic, neuroendocrine and neuroimmune pathways. This connection has been termed the microbiota–gut–brain (MGB) axis. Bacteria of the GM are able to produce neurotransmitters and other neurometabolites that are associated with depression [18]. These bacterial products can participate in the stimulation of central receptors; in the peripheral stimulation of nervous, immune and endocrine mediators; and in the epigenetic regulation of histone acetylation and DNA methylation [19]. Neurons of the enteric nervous system (ENS) interact directly with neurochemicals produced by the GM, thereby affecting the transmission of signals to the central nervous system (CNS). The disruption of various factors, though which the GM affects various systems and pathways in the host organism, has been commonly demonstrated during depression. These factors include oxidative stress and inflammation [20], tryptophan metabolism and the kynurenine pathway [21], mitochondrial dysfunction [22], neurotransmitters [23], brain plasticity and neurotrophic factors [24] and metabolic processes [25].

Numerous studies of the GM of patients with depression in comparison with healthy control groups showed dysbiotic differences including a decreased number of butyrate-producing beneficial bacteria, such as *Faecalibacterium prausnitzii*; *Roseburia intestinalis*; and genera *Ruminococcus*, *Coprococcus* and *Dialister*, and an increased number of inflammatory opportunistic bacteria from the Proteobacteria and Bacteroidetes phyla [26,27,28,29]. Gut dysbiosis (GD) affects the protective properties of the blood–brain barrier (BBB), including the modulation of permeability [30], and may lead to inflammation in the brain [31], which causes changes in mood and behavior [32,33]. The pathogenic effect of GD in many clinical studies has been associated with depressive and anxious behavior [34]. Changes in the population of certain species of the GM contribute to depression, and, on the other hand, a depressive state can induce modifications in the GM, which will lead to a more severe form of depression [35].

Identification of the GM’s bacteria and their metabolites, which can directly or indirectly influence the development of depression, is of great importance for both the creation of diagnostic systems for the disease and the selection of the strategies aimed at restoration of the normal functioning of the microbiota and, through this, restoring mental health. The search for approaches to the microbiota’s restoration in depression gained popularity in the last decade. Sufficient evidence confirms positive effects on depressive behavior from the application of the probiotic drugs that affect higher brain functions [36]. There is an increasing amount of literature considering various approaches as alternatives to pharmaceutical drugs used as antidepressants. These approaches also include diet and the use of dietary supplements such as probiotics, prebiotics and postbiotics [37]. Clinical improvements have already been shown in patients with neurological disorders after using such therapeutic approaches.

The review evaluates existing data on biomarkers of depression based on the taxonomy and functionality of the gut microbiota. The use of machine learning approaches for the analysis of a large-scale array of data on the gut microbiota of patients with depression for the selection of biomarkers is considered. Attention is paid to the commensal bacterium *Faecalibacterium prausnitzii* as an effective disease biomarker and diagnostic support tool and as a probiotic with antidepressant-like effects. The methods of weakening the manifestations of depression when using probiotics (both individual drugs and as adjuvants of antidepressants), prebiotics and postbiotics, including extracellular vesicles, are considered.

The presented analysis of published data on the study of the gut microbiota in depression may be useful for clinical practice and pharmacology.

## 2. Potential Mechanisms of Gut Microbiota Involvement in the Pathogenesis of Depression

It is currently known that the symbiotic interactions between the gut microbes and the host can affect mental health. The gut microorganisms are involved in interactions with the CNS, the ENS and the autonomic nervous system (ANS) through neuroimmune and neuroendocrine pathways via neural signals transmitted by the vagus nerve. Mucosal immunomodulation is carried out by the microbiota and its products, as well as chemical signals synthesized by the microbiota. In addition, the GM can control both the CNS and the ENS through the production and expression of neurotransmitters and neurotrophic factors, which modulate the sensory afferents of the intestine and the production of metabolites and maintain the integrity of the intestinal barrier and tight junctions. The GM participates in the maturation of microglia, neurogenesis and regulation of the expression form of neurotransmitter receptors in the brain and regulates the permeability of the BBB [30,38,39]. Through all these mechanisms, the GM can be involved in the pathogenesis of clinical depression. On the other hand, the effects of depression on the GM, regulated by stress, can change the release of neurotransmitters and other signaling molecules in the gut and affect the dysregulation of the immune response [35].

Understanding of the neuroimmune causes of depression and other stress-related mental disorders is growing, although the main mechanisms linking the immune, endocrine and neural systems with behavioral and psychological symptoms are not fully elucidated. A known risk factor for the development of MDD is the activation of the inflammatory response system. Basically, inflammation is caused by molecular structures of the pathogens, including bacterial lipopolysaccharides (LPSs), lipoproteins, flagellins and peptidoglycans, as well as endotoxin. Animal studies have shown that peripheral administration of LPSs leads to similar depressive behavior and an increased expression of IL-1β, TNF-α and nitric oxide synthase (iNOS) in the hippocampus and cerebral cortex [40]. The conducted image studies identified increased neuroinflammation in the brain of MDD patients compared with the control group [41]. The inflammatory effects of LPS spread from the periphery to the brain. The peripheral production of proinflammatory cytokines increases, and cytokines can reach the brain either through the transmission of signals mediated by macrophage-like cells or through the BBB [42].

Another type of inflammation is considered as “sterile inflammation” caused by psychological stress or molecular patterns associated with danger/damage (DAMPs), otherwise defined as alarms. These biomolecules are released as a result of tissue damage. GD can provoke autoimmune diseases due to inadequate post-translational modification of host proteins [43]. Intestinal microbes express a wide range of enzymes that are involved in post-translational modification of proteins (PTMP) in the gastrointestinal tract. Abnormal PTMP can lead to the formation of neoepitopes and subsequent autoimmunity. In all cases, bacteria are the source of receptor activation (PRRs), including Toll-like receptors (TLRs). For example, the activation of TLR4 by bacterial LPS triggers the so-called inflammasomes, receptors of the innate immune system, which additionally activate intracellular proinflammatory caspases, which leads to the release of proinflammatory cytokines. Cytokines cause depressive symptoms by affecting various processes related to emotions. Increased inflammatory signals can cause disorders of neurotransmitter metabolism, harm the normal state of nerves and disrupt brain regulation and signaling mechanisms in behavior and emotions [44]. Studies by Jin et al. showed that patients with a higher frequency of depressive symptoms produce more IL-6 compared to patients with a lower frequency [45]. A high level of nonspecific markers of inflammation was detected in patients with depression. Examples include acute phase protein, α1-antitrypsin, haptoglobin, fibrinogen and C-reactive protein [46].

GD can cause a violation of the regulation of inflammatory, stress (HPA) or neurotransmitter signaling pathways, leading to depression. For example, GD associated with a violation of the gastrointestinal barrier allows Gram-negative bacteria (Enterobacteriaceae) to enter the systemic circulation, activating immunoglobulin-mediated (IgA and IgM) immune responses to LPS, which leads to depression [47]. Experimental studies conducted on animal models over the past few years indicate a relationship between GD and depression-like behavior [48]. Several observational and clinical studies have also proved a direct correlation of depression with GD, confirming its role in the pathology of depression [49]. Various stresses are factors that can change the diversity and abundance of the gut microbiota and lead to dysbiosis. Such factors include sleep and its disorders. Sleep deprivation can exacerbate disturbances in the composition of the gut microbiota and disruption of circadian rhythms, which can lead to systemic inflammation and psychiatric disorders [50]. Stressors can disrupt the intestinal barrier, which becomes a source of systemic and central inflammatory responses and autoimmune diseases. Patients with MDD have elevated levels of 16S intestinal microbiota rDNA in peripheral blood, indicating bacterial translocation, which is accompanied by increased expression of TLR-4 RNA and protein in peripheral blood mononuclear cells, increased expression of NF-kB and an increased concentration of IL-6 [51].

Activation of the inflammatory pathway is characterized by the hyperproduction of reactive oxygen species (ROS) and reactive nitrogen species (RNS), followed by the damaged DNA, proteins, mitochondria and cell membranes [52]. Excessive ROS generation and the lack of efficient antioxidant response lead to inflammation, neurodegeneration, tissue damage and cell death [53]. The presence of oxidative and nitrosative stress in patients with depression is confirmed by the presence of high levels of lipid peroxidation products, such as malondialdehyde and 4-hydroxynonenal [54]. Depression is also characterized by decreased levels of endogenous antioxidants such as zinc, glutathione, coenzyme Q10, melatonin and vitamin E [55]. It has been shown that oxidative stress, along with inflammation, leads to the development of depression and related diseases [56]. There is increasing evidence that the GM can lead to both chronic inflammation and oxidative stress in the host’s tissues [57]. Germ-free (GF) mice show reduced activity of antioxidant enzymes (i.e., catalase, glutathione peroxidase and superoxide) [58]. During dysbiosis, the altered microbiota can stimulate NADPH oxidase [59] and NO synthesis [60], thus inducing OS.

Molecules produced by the GM can regulate functions of the gastrointestinal tract, such as nutritional, metabolic and immune responses, and can impact brain activity. Bacteria of the GM are the source of many neurotransmitters and neuroactive compounds, including gamma-aminobutyric acid (GABA), glutamate (Glu), serotonin (5-HT), dopamine (DA), norepinephrine (NE), histamine, acetylcholine and short-chain fatty acids (SCFAs) with anti-inflammatory properties and other properties [18]. Although most neurotransmitters produced by the gut microbiota cannot cross the BBB, they can stimulate the vagus nerve and alter the concentration of serotonin, GABA and glutamate within the brain in animals and humans [19]. Multiple studies have shown that changes in gut microbiota can alter brain levels of Glu [61]. Glutamatergic signaling has been linked to depression [62]. GABA neutralizes the action of glutamate. Low levels of GABA are associated with depression and mood disorders [63]. With increasing evidence of its role in the etiology of depressive disorders, Glu is rapidly becoming a new therapeutic target for the treatment of depressive disorders [63]. Stress, antibiotics and poor nutrition can cause dysfunction of the gut microbiome, which leads to disruptions in the work of neurotransmitters that are involved in depressive disorders along the MGA, which can be caused either by increased neuroinflammation or by a decrease in neuroplasticity and neurogenesis, and these effects are mediated by the vagus nerve or the HPA axis [64]. Other metabolites produced by the microbiota, such as LPS, lactate and B vitamins, are also associated with the pathogenesis of MDD [65,66]. The waste products of the GM can have a more pronounced effect on certain areas of the brain. For example, bifidobacterial enzyme arogenate dehydratase (ADT) is involved in the production of phenylalanine, which can pass through the BBB and be metabolized into the amino acid tyrosine, which can later be converted into DA and then into NE in the brain [67]. *Bifidobacterium infantis* increases the levels of circulating tryptophan, a precursor of 5-HT [68]. Many studies have reported that the compounds from tryptophan, tyrosine and purine pathways are expressed differently in patients with depression, which suggests that these metabolic components of the kynurenine pathway are possible mechanisms involved in the pathophysiology of MDD [69]. It has been demonstrated that during MDD there is an imbalance between the neuroprotective and neurotoxic branches of the kynurenine pathway with increased tryptophan metabolism towards the neurotoxic branch, which can lead to elevated glutamate neurotransmission [70]. Gut bacteria are involved in the production of melatonin, which, in addition to its circadian-rhythm-regulating role, also performs various immunoregulatory functions [71]. The number of butyrate-producing bacteria is significantly reduced in the GM of patients with depression [26,27,72]. The role of butyrate, which is a short-chain fatty acid, is multifunctional and consists in strengthening the protective barrier of the epithelium, reducing intestinal inflammation and others [73]. Some intestinal bacteria can interact with enteroendocrine cells (EECs) through their metabolites, controlling the production and release of neurotransmitters [72,74].

In patients with depression, the HPA axis is dysregulated, which leads to abnormally high levels of circulating corticotropin-releasing factor and cortisol. The HPA axis is one of the main axes of the endocrine system [75], which regulates the effectiveness and release of monoamine neurotransmitters, changes the content of the brain-derived neurotrophic factor (BDNF) and activates immune responses and systemic inflammation. It has been noted that in patients with depression, activation of the HPA axis can change the composition of the microbiota. Changes in the composition of the microbiota can lead to neuroendocrine diseases and increased intestinal permeability. Stress at an early age has a significant effect on the activation of the HPA axis [76]. The imbalance of the HPA axis caused by intestinal bacteria can affect the neuroendocrine system of the brain and cause anxiety-like behavioral phenotypes. In animal models, it was found that *B. infantis* normalizes the increased HPA axis response and reduces the symptoms of depression [77]. Thus, intestinal microorganisms play a significant role in the regulation of the HPA axis.

An important aspect of the MGB axis is the ability of the GM to control the maturation and function of glial cells. Abnormal morphology, immature phenotype and an increased amount of brain microglia were detected in GF mice, which was accompanied by a violation of innate immune responses [78]. MDD is also accompanied by abnormalities in non-neural glial cells [79]. Glial cells are in mutual communication with neurons, control various neuromodulating, homeostatic, metabolic and immune mechanisms and play a crucial role in neuroinflammatory mechanisms in MDD. These cells mediate the response of the CNS to systemic inflammation and psychological stress and can be a source of an inflammatory reaction in the CNS. Interactions between the gastrointestinal tract, increased intestinal permeability, GM and the cross-interaction of glia and neurons, as well as their role in the pathogenesis of the inflammatory hypothesis of MDD, were discussed in the review by Rudzki and Maes [80]. It was noted that the GM modulates glial functions by maintaining the permeability of the gut and BBB [30,81], controls the maturation and functions of microglia [78], affects the expression of genes involved in myelination [82], participates in the synthesis of aryl-carbohydrate receptor (AHR) ligands [83] and modulates serotonin and the kynurenine axis [84].

In addition to direct interaction with the CNS, the GM and its metabolites can regulate epigenetic processes, including DNA methylation, histone modification and the regulation of non-coding RNAs. Metabolites of the intestinal microbiota directly or indirectly affect the host genome by modulating its epigenome. Overcoming the BBB, metabolites produced by the GM participate in regulating the activity of epigenetic modulation enzymes. Various studies have revealed the potential role of epigenetics in the pathophysiology of depression [85]. Histone acetylation (H3 and H4) and methylation (H3) were observed along with chromatin modification after a mixture of the SCFAs was introduced to C57BL/6 mice [86]. It has been shown that DNA and histone methylation is regulated by the GM through the use of the enzyme L-methionine-S-adenosyl transferase (MAT) to synthesize S-adenosylmethionine (SAM) from methionine [87]. Folate, a methyl donor required for SAM synthesis, is produced by *Bifidobacterium* sp. and *Lactobacillus plantarum*. GD can affect SAM levels, ultimately altering the state of DNA and histone methylation [88]. Choline, produced by the GM, is an essential nutrient for healthy brain development and plays an important role in SAM production. Microbiota can cause an altered state of chromatin, which might result in host immune maturation [89].

Currently, it is known that some microRNAs (miRNAs) can regulate bacterial growth and gene transcription, as well as modulate the composition of the gut microbiota, which indicates the importance of miRNAs for gut and brain health. The review by Rosa et al. summarizes recent data on the potential role of microbiota and miRNAs in the neuropathology of depression and anxiety and their potential as treatment strategies [90]. miRNAs are single-stranded non-coding RNAs with an average length of 22 nucleotides that function as post-transcriptional regulators of gene expression, mainly through translational repression [91]. In GF mice, social interaction alters miRNA expression in the amygdala, confirming the link between a functioning microbiome and sociability, suggesting that miRNA may influence GM-modulated behavior [92]. Also, in animals with depleted microbiota, the expression of miRNA levels is altered [93], indicating the influence of microbiota on miRNA levels. It has been suggested that miRNAs are the most important signaling molecules involved in bidirectional microbiota–brain communication [94] since they can perform functional roles similar to hormones, affecting cells at a great distance from their original secretory sites [95]. miRNAs are an essential component of mouse and human feces and are necessary for maintaining normal intestinal microflora [96]. The microbiome regulates behavior and physiology under the influence of miRNAs [97]. Emerging data demonstrate that miRNA activity may play an important role in long-term changes related to the pathogenesis and treatment of depression [90]. miRNAs have already been recommended as pharmacological targets and biomarkers for the treatment and diagnosis of depression and anxiety [98].

**Figure 1:**
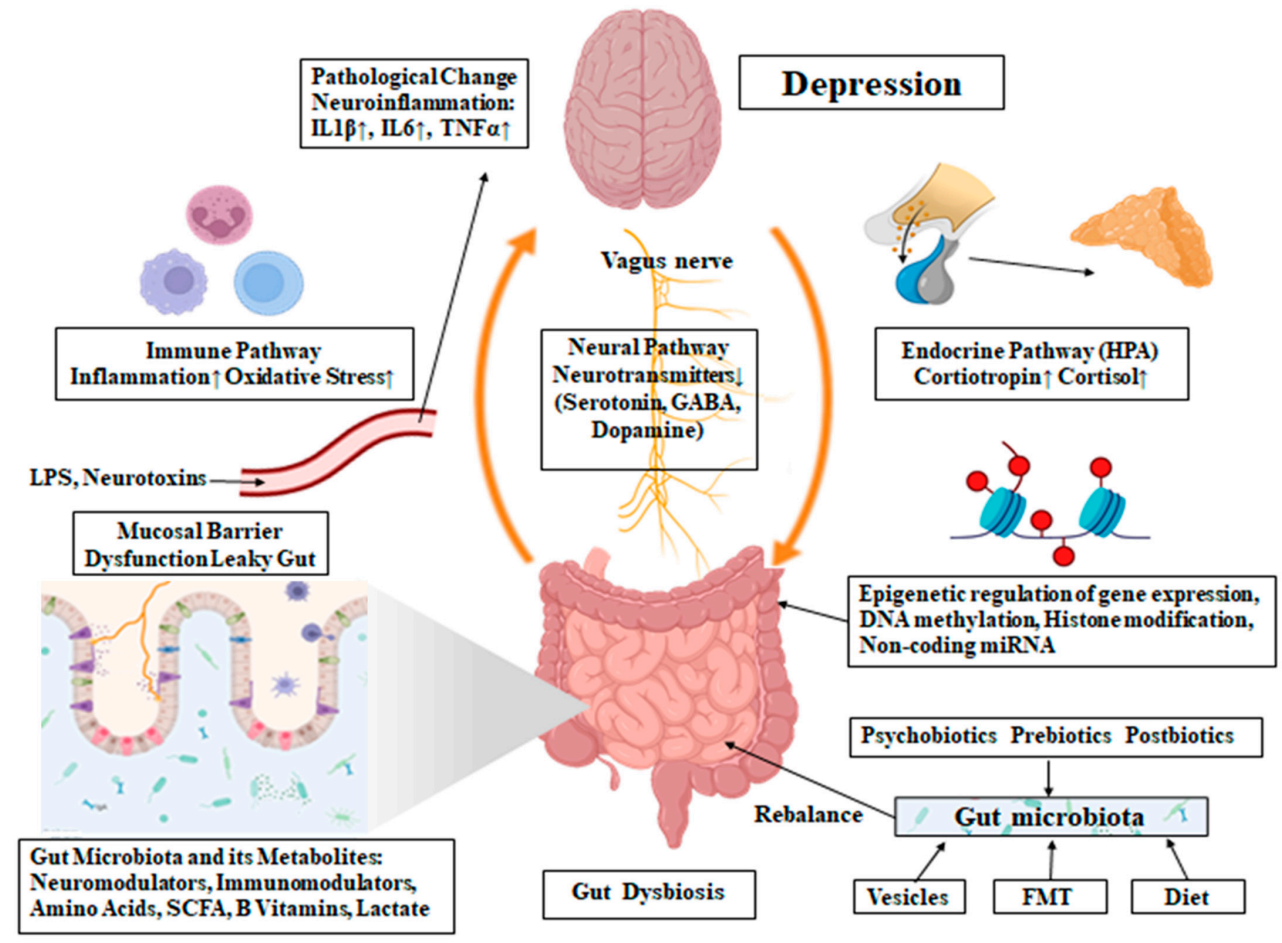
The mechanism of potential gut microbiota involvement in the pathophysiology of depression and its therapeutics.

The mechanism of potential involvement of the gut microbiota in the pathophysiology of depression is shown in Figure 1.

## 3. Using the Gut Microbiota to Search for Biomarkers of Depression

The human gut contains approximately one hundred trillion microbes of more than a thousand species that form a complex ecological community and play a key role in various biological processes, including health and disease [99,100]. The members of the GM are the main mediators of the body’s homeostasis. The diversity of the GM largely depends on various host factors, including diet, human lifestyle, age and environmental factors [101]. Normally, the GM is characterized by a high diversity and abundance of microbial populations, and this condition is known as “eubiosis”. During life, a wide range of factors, including poor nutrition, sleep disorders, obvious pathological conditions, drug abuse, pharmacological therapy and many others, can change the diversity and abundance of the microbiota, leading to a state of “dysbiosis”. Dysbiosis is accompanied by a decrease in the number of beneficial butyrate-producing bacteria, such as *F. prausnitzii* and *Roseburia* sp., and an increase in the number of opportunistic bacteria, such as *Clostridium symbiosum* and *Escherichia coli* [102]. Today, it is known that gut dysbiosis can cause or exacerbate various neuropsychiatric disorders [103]. Patients with such disorders often have gastrointestinal symptoms [104].

Currently, the GM is considered as an important factor in the occurrence and maintenance of depression. Numerous studies have shown differences in the composition of the GM in people with depression in comparison with a healthy control group. The abundance levels for some major genera of bacteria in the microbiota differed significantly between MDD and healthy control groups. Studies of the GM, mainly using 16S rRNA sequencing (more than 90% of the included studies), showed increased levels of representatives of the genera *Blautia*, *Escherichia-Shigella*, *Ruminococcus* and decreased levels of the genera *Faecalibacterium*, *Prevotella*, *Roseburia*, *Agathobacter*, *Bifidobacterium*, *Lachnospiraceae*, *Butyricicoccus*, *Lactobacillus* in patients with MDD in comparison with the control group [29,105,106]. The taxonomic meta-analysis of 16S rRNA sequences from 1827 samples from eight different cohorts of different populations showed enrichment with bacteria *Bacteroidetes*, *Parabacteroides*, *Barnesiella* and *Bacteroides* and, at the species level, *Bacteroides distasonis*, *Bacteroides vulgatus*, *Alistipes inops* and *Bacteroides massiliensis*, as well as depletion of *Firmicutes*, *Dialister* and *Bacteroides plebeius*, in the microbiota associated with depression [107]. Unfortunately, the profile of microbiomes during depression differs greatly in various studies, which is probably due to biological differences across the cohorts and methodological differences in the processing of samples. Also, patients from different countries have different genetics and dietary patterns, which significantly affect the gut microbial composition.

The different results obtained in various studies conducted mainly in Europe and Asia may also be due to differences in the antidepressants used by patients. Changes in the gut microbiota after taking antidepressants have previously been demonstrated in clinical studies of patients with depression [108]. An in vitro approach using bacterial cultures of *Bifidobacterium animalis*, *Bacteroides fragilis* and *F. prausnitzii* demonstrated that several antidepressants exhibit significant antimicrobial activity against commensal bacteria. At the same time, desipramine and aripiprazole were the most inhibitory [109]. The antidepressants fluoxetine, escitalopram, venlafaxine and duloxetine have also been shown to reduce the richness of microbial communities [110]. Treatment with psychotropic medications (psycho-pharmacomicrobiomics) also was associated with altered gut microbiome composition [111].

Using next-generation sequencing (NGS) technologies, which allow the study of whole genomic DNA, research of the GM of patients with depression showed similar taxonomic differences, obtained using 16S rRNA sequencing. There was a decrease in the level of butyric acid producers (even at the species level), such as *F. prausnitzii*, *Lachnospira eligens*, *Roseburia hominis*, *Roseburia intestinalis* [27], *Dialister*, *Coprococcus* spp. [26], and an increase in bacteria with LPS that cause inflammation, *Escherichia coli* and *Ruthenibacterium lactatiformans* [27]. In general, taxonomic changes in the microbiota of patients with depression are associated with a proinflammatory condition, a decrease in the number of anti-inflammatory bacteria (*Faecalibacterium*, *Firmicutes*) and an increase in the number of inflammatory *Alistipes*, *Flavonifractor*, Bacteroidetes, Gammaproteobacteria [27]. In the majority of studies, the *Faecalibacterium* taxon had lower abundance in the GM of patients with depression [106].

The gut microbiome consists of more than 3 million genes involved in the production of thousands of metabolites and, consequently, affecting many aspects of human health [99]. Various microbial metabolites and compounds have an important impact on human mental health due to the manifestation of neuromodulating, immunomodulating, anti-inflammatory and antioxidant properties [112]. These effects will depend on the composition of the microbiota and on the diet. Changes in the composition of the microbiota of patients with depression can be associated with differences in levels of bacterial metabolites. In the GM of patients with depression, the number of bacteria producing SCFAs is reduced [26,27,29]. Stool samples of patients with MDD contain lower levels of total SCFAs compared to a healthy control group [113]. In the pathophysiology of depression, SCFAs affect epigenetic mechanisms; participate in reducing the production of proinflammatory cytokines, the maturation of microglia and BDNF production; strengthen the protective barrier of the epithelium; and stimulate the vagus nerve [114]. The importance of SCFAs is further confirmed by various clinical trials. For example, patients with MDD experienced a decrease in the symptoms of depression after administration of probiotics that produce SCFAs, and an improvement in mood and cognitive abilities was reported for healthy people [115]. The study by Liang et al. showed increased degradation of L-glutamine and reduced biosynthesis of L-glutamate and L-isoleucine in microbiomes associated with depression [107]. In a study, Xie et al. [116] observed a significant decrease in arginine levels in the plasma of MDD patients and found that arginine and its metabolites citrulline, ornithine and proline were negatively correlated with depression severity. These findings suggest that the decline in arginine and proline metabolism contributes to the progression of depression. The lower fecal levels of L-lysine and N-ε-acetyl lysine may predict the effectiveness of therapy in patients with depression and anxiety. Symptoms improved as the levels of these markers increased [117]. The involvement of various bacterial metabolites in the pathogenesis of depression is considered in various reviews [18,28]. A comparative study of shotgun metagenomic data using a gene catalog of the key bacterial enzymes relevant to depression [18] revealed a decrease in the number of metabolic genes involved in the production of arginine, asparagine, conjugated linoleic acid, GLU, glutamine, melatonin and spermidine, in correlation with a decrease in the number of *F. prausnitzii* in the GM of patients with depression [27], and revealed the potential role of the production GABA in the development of depression [26]. When *Faecalibacterium* species and *Prevotella copri* were less abundant, alterations were observed in glutamate metabolism [118]. *F. prausnitzii* metabolites with anti-inflammatory effects [119] are of great interest. It has been found that different ratios of strains and phylotypes of *Faecalibacterium* are associated with several diseases [120]. *Faecalibacterium* could serve as an effective disease biomarker and diagnostic support tool. The investigation of the relationship between the diversity and composition of the fecal microbiome of 1054 Rotterdam patients with symptoms of depression revealed an association of thirteen taxa, *Eggerthella*, *Subdoligranulum*, *Coprococcus*, *Sellimonas*, *Lachnoclostridium*, *Hungatella,* Ruminococcaceae (UCG002, UCG003 and UCG005), Lachnospiraceae (UCG001), *Eubacterium ventriosum Ruminococcus gauvreauii* and the family of Ruminococcaceae, which are involved in the synthesis of glutamate, butyrate, serotonin and GABA [121].

**Figure 2:**
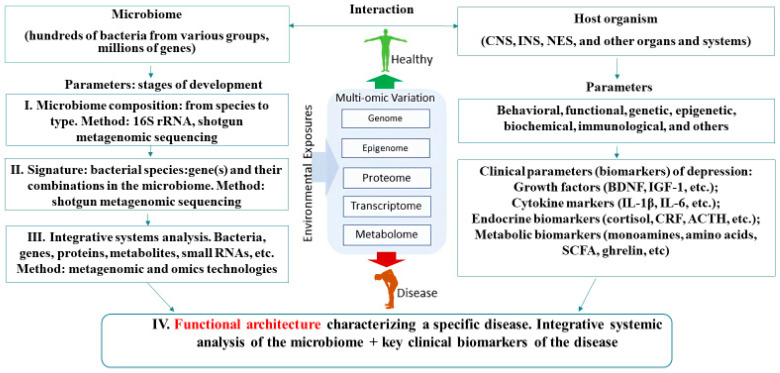
Evolution of parameters characterizing the microbiome–host organism system (superorganism).

Thus, in an analysis of the published data, there is a significant heterogeneity in the results obtained in the study of GM patients with depression from different populations conducted in various laboratories around the world. There is no clear understanding of which common gut bacteria and their metabolites can be used as biomarkers of depression. However, the search for microbiome-based diagnostic biomarkers for depression will continue as the methodological framework for microbiome studies improves and is standardized. Today, known changes in taxonomy and metabolic pathways correlating with depression can be used as biomarkers only for patients from a specific population on which the research was conducted. More attention should be paid to the study of genes in the gut metagenomes involved in the metabolism and synthesis of compounds with neuromodulatory and anti-inflammatory activities associated with diet, which correlate with depression. The microbiota has the ability to specifically modify biologically active food ingredients. For planning subsequent research on the gut microbiome and its role in the diagnosis, prevention and treatment of depressive disorders, it is important to analyze the evolution of biomarker parameters describing the microbiome system of the host organism. The sequence of events is presented in Figure 2. For many years, the search for biomarkers of depression at the level of the gut microbiome and other human systems and organs (CNS, ENS, NES, etc.) has been conducted either practically in parallel or rarely personally. The signature approach has allowed us to identify genes/bacteria and potentially their products capable of enhancing or complementing the synthesis of neurotransmitters and their precursors synthesized by cells. In the next stage, an integrating systemic analysis will be conducted using metagenomic and omics technologies to identify specific species and phylogroups of bacteria responsible for the synthesis of low-molecular-weight metabolites, proteins, enzymes and small RNAs playing a key role in maintaining the organism in a state of positive homeostasis.

An important step towards a new level of research will be the introduction of a new concept—the functional architecture of the disease [122]. This concept includes an integrating systemic analysis of the microbiome plus key clinical biomarkers of the disease. In the future, the epigenetic biomarkers accompanying the disease will be included in this system. Further advancement in this direction is unthinkable without further digitization of the microbiome and the use of artificial intelligence technologies.

## 4. Machine Learning Approaches for the Diagnosis of Depression Based on the Biomarkers of the Gut Microbiota

Modern metagenomic research can be conducted using two key types of data: 16S rRNA genes and/or the whole genomic material of microorganisms from the microbiota (whole metagenome). The first data are primarily used for taxonomic analysis, and the latter can be utilized for both taxonomic and functional analyses.

In order to uncover the dependencies between the traits of microbiota, such as the abundance of taxonomic units, specific genes and/or metabolic pathways, and various conditions of the subject (disease, body mass index, geographic origination, diet, etc.), specific methodologies are required. Most commonly they include traditional correlation tests, like Spearman or Kendall rank tests, for the examination of correlations with one condition and linear regression models implemented in bioinformatics packages, such as MaAsLin2, for correlations with several traits [123]. Specific taxa and/or genes that describe major changes in the gut microbiota during a disease, for example, depression, can further be treated as indicators of the disease, i.e., biomarkers [124]. Metagenomic signatures that describe both the taxonomic composition of the microbiota and the genes comprising the metagenome can also be used as potential biomarkers [27].

Modern computational algorithms have introduced a whole new group of methods, the machine learning (ML) approaches. The general idea of many ML algorithms is to train a model based on a list of parameters and a special training dataset and then use it for the analysis of a general dataset. One of the most important parts of application of the ML is the assembly of an appropriate training dataset, which avoids such problems as biases, batch effect, noisiness and dataset size. Metagenomic datasets include a colossal number of parameters (dimensions) by which the samples can be described, for example, abundances of certain taxa or genes. In order to reduce the number of dimensions in the dataset, it is common to highlight the most important features, which can be used for classification and prediction, using several methods: principal component analysis (PCA), statistical analysis, deep learning, etc. [125]. The most popular ML algorithms that have been applied in metagenomic studies include linear and Gaussian support vector machine (SVM), naïve Bayesian classification, random forest, logistic regression (LR) and various deep learning algorithms [126,127]. Random forest classification is one of the most widely used algorithms in metagenomics thanks to its ability to work with relatively small datasets while delivering stable results [128]. With the help of various ML approaches, researchers can solve the tasks of taxonomic characterization, sample clusterization and correlation of microbiota composition with various diseases [129].

Several studies utilizing ML methods for analyzing the human gut microbiota during depression have been conducted to date. In the large gut microbiome-wide association study by Radjabzadeh et al., naive Bayesian classifier was used for the taxonomic classification of more than 2500 16S rRNA samples obtained from patients with depression and healthy controls from Rotterdam and Amsterdam [121], which resulted in the identification of 12 genera significantly associated with the disease, 10 of which belonged to the families Ruminococcaceae and Lachnospiraceae. Further regression analysis using Breiman’s random forest algorithm and Mendelian randomization allowed for a description of the link between the major depressive disorder and the increased abundance of the genus *Eggerthella*. Another 16S rRNA-based study attempted to utilize the support vector machine classifier algorithm for a multi-cohort analysis, which included 1827 samples from eight different cohorts, in order to describe depression-associated taxonomic markers [107]. As a result, the researchers observed a significant increase in the abundance of Bacteroidetes, *Parabacteroides*, *Barnesiella*, *Bacteroides*, *Alistipes inops* and *Bacteroides massiliensis*, along with depletion in Firmicutes, *Dialister* and *Bacteroides plebeius*. The implication of using Dirichlet Multinomial Mixtures also allowed the researchers to establish a cross-cohort cluster of taxa in the gut microbiota associated with depression. This cluster was represented by a lower abundance of *Escherichia*-*Shigella* and a higher abundance of *Faecalibacterium*, Oscillospiraceae UCG 002, *Ruminococcus* and Christensenellaceae R.7 group.

ML algorithms can also be used for the whole metagenome data, which not only describe the taxonomic abundance but also include information about the genes and metabolic pathways. In a recent work, it was demonstrated how the ML approach can be applied to develop a toolkit for in silico diagnosis of depressive disorder based on the biomarkers of the gut microbiota [130]. The classifications were made based on metagenomic signatures. The resulting list of the identified biomarkers that characterize the condition of the gut microbiota during depression included genes involved in the production of acetic and butyric SCFAs, arginine, asparagine, glutamate, inositol and spermidine. It is notable that many of these genes were identified in the genomes of species *F. prausnitzii*. The utilized methods included the random forest, elastic net and the ‘You Only Look Once’ (YOLO) method [131]. YOLO is an algorithm that is normally used in image recognition tasks. Since the results of signature metagenomic analysis can be pictured as a heatmap, the application of such an approach was convenient and allowed for the high average prediction accuracy of 94.5% during validation.

## 5. Approaches to Restoration of the Gut Microbiota in Depression

Today, the development of alternative approaches targeting gut dysbiosis in neuropsychiatric diseases (NPDs) is gaining popularity. A growing number of studies support the preclinical results and advise reestablishing a healthy gut by developing a well-balanced microbial composition and diversity through dietary modifications, fasting, calorie restriction and using supplements such as probiotics, prebiotics, postbiotics or FMT.

### 5.1. Probiotics

Advances in our understanding of the role of the microbiota in the onset and progression of depression have led to the realization that utilizing gut microbiota bacteria and their metabolites as probiotics may offer a promising alternative. The effectiveness of probiotics in alleviating depressive symptoms has been demonstrated in numerous laboratory animal experiments [43,132,133]. Probiotics tailored for the treatment of depression have also been investigated in human studies. The results of these studies are detailed in articles focusing on specific probiotics, as well as in systematic reviews and meta-analyses [43,76,133,134,135,136,137,138,139]. This concept has given rise to the term ‘psychobiotics’, which encompasses probiotics, prebiotics and all microbiota-targeted interventions that positively impact mental health [138,140,141,142]. The majority of these studies primarily utilized various strains of lactobacilli and bifidobacteria as potential antidepressant probiotics. Probiotics occasionally incorporate bacteria from other genera such as *Streptococcus thermophilus*, *Lactococcus lactis* and *Bacillus*. In most cases, the administration of specific probiotic strains resulted in a significant reduction in depressive symptoms compared to a placebo.

Probiotics can exist as single-species monocultures [143] or multi-species mixtures [144], and many also contain prebiotics (oligosaccharides, inulin and others), classifying them as synbiotics [145]. The aforementioned studies have observed that probiotics not only reduce depression and anxiety symptoms, as measured by validated questionnaires, but also lead to decreased feelings of anger/hostility, obsessive–compulsive behaviors and paranoid ideation. Probiotics contribute to the normalization of the microbiota composition and an increase in its diversity [146,147]. Furthermore, probiotic use has been associated with a decrease in cortisol levels in plasma and saliva [148,149,150] and urine levels of methylamines and aromatic amino acid metabolites [143]. It has also led to an enhancement of the serotonin pathway [150,151]. Additionally, probiotic use has been linked to a decrease in proinflammatory cytokines and an increase in anti-inflammatory IL-10 in plasma [147,150]. It has also been associated with a reduction in plasma markers of inflammation (myeloperoxidase, C-reactive protein) [152], as well as an increase in antioxidant markers (total glutathione level) and elevations in BDNF levels in the blood and serum [146]. Additionally, probiotic use has been linked to increased electroencephalographic neural activity in the prefrontal cortex [148].

The efficacy of probiotics varies depending on several factors. The antidepressant effects, like any probiotic activity, are specific to particular species and strains of bacteria, making the outcome dependent on the characteristics of the bacterial strain in use. The form of the probiotic is also crucial, with solid formulations recommended over fermented milk and sachets [153]. Multi-strain probiotics often demonstrate superior therapeutic efficacy compared to single-strain counterparts, while single-species probiotics are believed to facilitate gut colonization [135,154]. The effectiveness of treatment increases with higher doses and longer durations. For effective antidepressant therapy, probiotics should be administered at a dose higher than 10^9^, preferably 10^10^, CFU/day for at least 8 weeks [137].

The outcome of probiotic action is also influenced by the host organism, specifically factors such as age, gender and genetic makeup [135,155]. Multi-strain probiotics have demonstrated the most pronounced antidepressant properties in individuals with an SNP mutation in the *IL-1β* gene [156]. Different cohorts of people may respond differently to the effects of probiotics. For instance, the probiotic Cerebiome (*Lactobacillus helveticus* R0052 and *Bifidobacterium longum* R0175) did not exhibit antidepressant properties in one study [157] but did in several others [158,159]. When comparing the effects of probiotics on healthy and depressed individuals, probiotics mainly showed significant antidepressant-like effects in cases of clinical depression [135,160,161,162]. The degree of depression and anxiety is assessed using verbal tests, and the results of determining the effectiveness of probiotics may vary depending on the specific test used [149,163].

Regrettably, there is an insufficiency of long-term observations regarding the utilization of probiotics. Some studies have shown that, upon discontinuation of their use, the effects on depression and anxiety symptoms may either persist for several weeks [143] or diminish [148].

To ascertain the precise mechanism of action of probiotics and to formulate pharmaceutical postbiotic products based on them, it is imperative to pinpoint the specific compounds responsible for their activity. While numerous cellular components and metabolites of bacteria are known to be bioactive compounds [8,137], only some of them have been unequivocally linked to antidepressant activity. Notably, exopolysaccharides (likely associated with the activity of certain heat-inactivated probiotics), GABA, SCFA (particularly butyrate), H_2_O_2_ and carboxyesterase (an enzyme converting albiflorin to benzoic acid) have been identified as having such associations [134]. Additionally, researchers have recently turned their attention to the activity of extracellular vesicles (discussed in the subsequent section on postbiotics).

Our understanding of probiotic mechanisms has primarily been gleaned from preclinical and in vitro data. A synthesis of research findings suggests that probiotics influence the composition of the microbiota, avert gut dysbiosis, reduce gut inflammation, reinforce the intestinal barrier, modulate the central GABAergic system, mitigate HPA axis overactivity, inhibit the activation of indoleamine 2,3-dioxygenase (an enzyme crucial in immune cells that catabolizes tryptophan into kynurenine), impact the central DA system and upregulate the central 5-HT-BDNF system [154,164]. The specific effects of a given probiotic are directed toward one or more of these targets. Consequently, if a probiotic primarily modulates the composition of the gut microbiota, its efficacy may vary among different demographic cohorts. This variability may explain why certain probiotics exhibit greater effectiveness in individuals with depression compared to their healthy counterparts [135,154].

The term probiotic refers mostly to a food or dietary supplement, and much less frequently to a drug or medicinal product. To identify probiotics that are specifically drugs, the term Live Biotherapeutic Products (LBPs) was proposed [165,166]. The US Food and Drug Administration defined LBPs as biological products containing live organisms, applicable to the prevention, treatment or cure of a human disease or condition. At present, no definitive clinical recommendations can be established for any specific antidepressant probiotic. Nevertheless, a plethora of commercially available probiotic products are designed to alleviate stress, anxiety and depression symptoms [133,137,142,167] and may be potential LBPs [168]. Among these, several have been subjected to clinical trials and extensive investigation, including the following:

“Cerebiome” (formerly “Probio’Stick^®^”) (Lallemand Health Solutions Inc. Mirabel, Canada): Comprising *L. helveticus* R0052 and *B. longum* R0175, this product has been the subject of investigation in five clinical trials and nine preclinical studies.

“Ecologic Barrier” (Winclove Probiotics, Amsterdam, The Netherlands): It encompasses nine strains of bifidobacteria, lactobacilli, and *L. lactis*.

“OMNi-BiOTiC^®^ Stress Repair” (Institut Allergosan, Graz, Austria): It features nine strains of bifidobacteria and lactobacilli, along with B group vitamins.

Despite the consistent demonstration of antidepressant effects by these formulations, they cannot be universally applicable. As mentioned earlier, the “Cerebiome” preparation exhibited antidepressant activity in specific demographic cohorts but not in others. The same holds true for the “Ecologic Barrier” formulation [133].

The use of probiotics in monotherapy often, but not always, leads to a reduction in depression symptoms. More promising and effective is the use of probiotics for the treatment of MDD in combination with antidepressants. There are several studies where probiotics are used as adjuvants to treat patients with major depression [145,158,169,170,171,172,173] and anxiety disorders [174]. Another article is focused on the evaluation of depressive symptoms in patients with bipolar disorder [175]. The earliest article was published in 2016, suggesting that research on combination therapy with probiotics in conjunction with antidepressants is a new direction. In all studies, the experimental group received psychotropic medications in combination with a probiotic supplement, and the control group received antidepressants alone.

Among the antidepressants used, serotonin reuptake inhibitors (SSRIs) were used in all depression trials; Kazemi et al. [158] used amitriptyline (a tricyclic antidepressant) in addition to SSRIs. Zhang et al. [175] used lithium carbonate, lamotrigine, quetiapine and lurasidone. Some antipsychotics and antidepressants have antibacterial effects, so it is important to consider the specific medications used in each study. SSRIs have been found to differ in their degree of inhibition of bacterial growth, with sertraline and fluoxetine having the strongest antimicrobial activity, followed by paroxetine and fluvoxamine, and then escitalopram and citalopram [176]. Depressive state was assessed using the Hamilton Depression Rating Scale [145,170,171,172,175]. Akkasheh et al. [169] and Kazemi et al. [158] assessed depressive symptoms using the Beck Depression Inventory; this scale was also used by Miyaoka et al. [170]. Additional Symptom Checklist-90 and Perceived Stress Scale-10 (PSS-10) were used by Rudzki et al. [171]. Anxiety was assessed using the Hamilton Anxiety Scale [174]. Depression was mild to moderate in all studies except that of Miyaoka et al., where resistant depression was considered [170]. Different bacteria strains were used: *Lactobacillus acidophilus*, *Lacticaseibacillus casei*, *Bifidobacterium bifidum* [169]; *Lactiplantibacillus plantarum* [171]; *L. helveticus*, *B. longum* [158]; *Clostridium butyricum* [170]; *B. longum*, *B. bifidum*, *Bifidobacterium lactis*, *L. acidophilus* [174]; the synbiotic Familact H^®^ containing seven strains of lactobacilli, bifidobacterial and streptococci [145]; Bac-Set Forte, a multi-strain probiotic containing 14 strains of lactobacilli, bifidobacteria, streptococci, lactococci [172]; *Bifidobacterium*, *Lactobacillus*, *Enterococcus* and *Bacillus cereus* [173]; *B. lactis* [175].

Most articles showed that adjuvant probiotic therapy was more effective than antidepressant therapy separately in reducing depression symptoms. One study [171] found no significant effect on symptom severity in depressed patients, but found that adjuvant probiotic therapy correlated with improved cognitive performance. Shi et al. [172] did not assess depressive symptoms at the end of the experiment, but noted that combination therapy improves cognitive functions. In all studies, despite the difference in research conditions, the use of a combination of a probiotic and an antidepressant gave a positive effect compared to the control.

In addition to symptomatic evaluation, various parameters in patients’ blood were evaluated. In the study of Rudzki et al. [171], the only one where no reduction in the severity of depressive symptoms was observed, a reduction in kynurenine concentrations was found. Kazemi et al. [158] studied the effects of probiotics on tryptophan metabolism. The microbiota indirectly affects serotonin synthesis by reducing the activity of the enzymes responsible for tryptophan degradation through the kynurenine pathway. The branched-chain amino acids (leucine, isoleucine and valine) compete with tryptophan for transporters. Because circulating levels of tryptophan do not directly reflect the availability of tryptophan in the brain, it is better to measure serum levels of the ratio of tryptophan to branched-chain amino acids rather than total serum tryptophan concentration. As a result, the authors noted an increase in the tryptophan/isoleucine ratio before and after treatment in the group of patients receiving the probiotic [158] The largest number of biochemical parameters was assessed in the work of Arifdjanova et al. [172]. The authors evaluated monoamine and proinflammatory system components, and also NO. Due to probiotic therapy in combination with an antidepressant, a statistically significant decrease in cortisol, dopamine, proinflammatory cytokines (IL-6, TNF-alpha) and NO levels was observed in patients receiving combined therapy compared to a control group. The use of probiotics led to a more pronounced improvement of nervous–immune–endocrine parameters in the studied patients, with a following reduction in symptoms of depressive disorders. Shi et al. noted a significant decrease in the cortisol and interleukin-1 levels in the study group compared to those in the control group [173]. In Akkasheh et al.’s work, combined therapy had beneficial effects on insulin, homeostasis model assessment of insulin resistance, hs-CRP concentrations and glutathione concentrations [169].

A series of researchers conducted a comprehensive study of depressed patients receiving a probiotic (Vivomixx, eight strains of lactobacilli, bifidobacteria and *S. thermophilus*) along with conventional antidepressant treatment. Patients who received the probiotic maintained alfa microbial diversity in the gut and had structural and functional changes in the brain correlating with a reduction in depressive symptoms and remediated hippocampus function, in contrast to patients who received a placebo [177,178,179].

Thus, the findings demonstrate the potential of combined therapy with an antidepressant and a probiotic. In the study of the mechanisms of action of probiotics, the authors of the articles were limited to the effect on metabolism and the monoamine system, and one study was focused on the investigation of inflammatory factors. However, the effects of the microbiota on humans are not limited to these factors. The interaction of antidepressants and probiotics and the identification of synergistic effects is also an open question. Further research should be devoted to this problem in order to identify the most suitable probiotic and traditional drug pairing for the therapy of depression.

To obtain effective and stable probiotic drugs, different biotechnological methods of microbial encapsulation are needed that improve survival rate during processing, storage and GIT transit (spray drying, freeze-drying, extrusion, emulsion-based techniques and others) [180].

The effects of probiotics on patients with depression in double-blind, placebo-controlled trials are presented in Table 1.

Several bacteria that constitute the core population of the healthy human gut microbiota hold promise as next-generation probiotics and prebiotics. Intervention with *Akkermansia municiphila* significantly improved depressive-like behavior in mice and rectified aberrations in depression-related molecular markers (corticosterone, dopamine, and BDNF) [181,182]. The protein *A. muciniphila* Amuc_1100, a 32 kDa pili-like outer membrane protein, exhibits activity akin to that of *A. municiphila* cells [183,184]. Two isolated strains of *F. prausnitzii* have demonstrated improvements in cognitive impairment within mouse models of Alzheimer’s disease [185]. *F. prausnitzii* ATCC 27766 has manifested anxiolytic and antidepressant-like effects and reversed the effects of chronic unpredictable mild stress in rats [186]. *C. butyricum*, whether as a monopreparation or in combination with antidepressants, has been shown to alleviate manifestations of depressive states in mice [43]. The strain *C. butyricum* CBM588, in conjunction with antidepressants, has reduced median scores in the treatment of patients with resistant major depressive disorder (MDD) [170]. Certain strains of *Enterococcus faecalis*, known for their beneficial effects in the dairy industry as probiotics and starter cultures, can prevent depression-like behavior in mice. However, other strains of *E. faecalis* are implicated in nosocomial infections [43]. The overwhelming majority of gut microbiota bacteria are obligate anaerobes, posing challenges for their cultivation and the development of probiotics. Pioneering techniques to adapt anaerobes to tolerate oxygen exposure may facilitate widespread utilization of these strictly anaerobic bacteria as probiotics [187].

### 5.2. Prebiotics, Postbiotics, Extracellular Vesicles and FMT

In addition to probiotics, prebiotics also exhibit biological activity. Prebiotics are substrates (typically fiber, galactooligosaccharides (GOSs), polyphenols and inulin, compounds from vegetables, herbs and plants) that confer a positive health effect when metabolized by commensal microbes but do not include the microbes themselves. In some studies on rodents, GOS prebiotics have been shown to modulate anxiety and depressive-like behavior and elevate brain BDNF in the hippocampus [188]. In a clinical trial, trans-GOS prebiotics reduced anxiety scores in people with irritable bowel syndrome [189]. Four weeks of GOS treatment reduced self-reported anxiety scores in highly anxious participants [190]. A meta-analysis showed that the use of polyunsaturated fatty acids alleviates depressive symptoms in humans [191,192]. Water-soluble cellulose acetate, which is fermented by intestinal bacteria and increases the production of short-chain fatty acids and GABA in the human gut, has the potential to be a prebiotic [193]. However, in the majority of studies, prebiotics used as monopreparations did not influence the manifestation of depression symptoms in humans, unlike probiotics and synbiotics [76,88,136,138,158]. Synbiotics have been noted for their greater effectiveness in reducing depression symptoms compared to probiotics [162].

Although probiotic bacteria demonstrate the potential to prevent and alleviate various diseases, they are not without risks. Probiotics, being living organisms, can trigger inflammatory conditions, especially in children and immunocompromised individuals. In contrast, postbiotics are regarded as safer alternatives. Postbiotics encompass inanimate microorganisms and/or their components, offering health benefits to the host [166]. These components include inactivated bacterial cells, cell-free culture fluid, cellular constituents, cell lysate and metabolites produced by microorganisms. Postbiotics exhibit antimicrobial, antioxidant and immunomodulatory properties, contributing to the management of numerous diseases. Additionally, they find application in the food industry as functional additives, enhancing taste and preserving product quality [194]. Several inactivated strains of lactobacilli, bifidobacteria and enterococci have demonstrated antidepressant effects in both preclinical and clinical studies when used as postbiotics [195]. Notable postbiotic candidates with potential antidepressant properties include biologically active substances generated by gut microbiota bacteria, although only a subset of them have proven the ability to alleviate depressive symptoms when consumed. These substances encompass SCFAs (with a particular emphasis on butyrate) [196] and folate [197]. The protein Amuc from *A. municiphila* also qualifies as a postbiotic, as its intervention has proven effective in mitigating stress-induced depression-like behavior in mice. It achieves this by improving gut microbiota composition, elevating BDNF levels, and suppressing neuroinflammation, all while remaining stable even after pasteurization [183,184].

A promising subset of postbiotics falls under the category of extracellular vesicles (EVs). EVs are nanoscale vesicles naturally released from cells and enclosed by a lipid bilayer. Their cargo comprises proteins, lipids, polysaccharides, phages, DNA, RNA and microRNA. Unlike living microorganisms, EVs cannot replicate, making them safer. EVs, secreted by various eukaryotic and prokaryotic cells, are found in numerous biofluids. They possess biocompatibility, low immunogenicity, stability and the ability to traverse biological barriers, including the blood–brain barrier (BBB). EVs release their contents to target cells through short-distance and long-distance mechanisms, thereby regulating target cell activity. In humans, EVs have been implicated in various brain-related functions and may play a role in the pathogenesis of psychiatric and neurodegenerative disorders, including depression. They are currently under investigation for both diagnostic and therapeutic purposes in MDD [198]. Bacterial EVs perform a wide variety of functions: exporting misfolded proteins, peptidoglycan fragments, etc., from cells; binding and transporting cytosolic metabolites on the outer surface of EVs; binding and neutralizing phages, antibiotics and biologically active peptides; DNA transfer; delivery of bioactive compounds [199].

There is evidence of EVs’ presence in probiotic bacteria, including lactobacilli, bifidobacteria and lactococci. Probiotic EVs harbor numerous biologically active substances [200], exhibiting immunomodulatory activity and fortifying the integrity of the intestinal barrier [201]. In animal studies, intraperitoneal injection of *L. plantarum* KCTC 11401BP EVs has been shown to restore the expression levels of BDNF in the hippocampus, reducing depressive-like behaviors in mice exposed to stress, both during the stress induction phase and on days 29–30 post-stress [202]. Similar effects, though not identical, have been observed with EVs derived from *Bacillus subtilis* and *A. muciniphila* [203]. EVs from *L. plantarum* reduced brain damage and improved neurological function in mice following a stroke. The mechanism of this protective effect involved the regulation of a specific microRNA, miR-101a-3p [204]. EVs from potential probiotics such as *A. muciniphila* and *F. prausnitzii* have been found to affect serotonin signaling/metabolism in Caco-2 cells, potentially positioning them as postbiotics for the treatment of serotonin-related disorders, including depression. *A. muciniphila* and its EVs increased the mRNA expression of genes involved in serotonin signaling/metabolism in the colon and hippocampus of mice and may be considered as new therapeutic strategies to ameliorate serotonin-related disorders [205].

Bacterial EMs communicate at the inter-kingdom level to affect the gut–brain axis. They are likely one of the main modes of action of probiotics. The most likely mechanism of EV distribution to the CNS is by crossing the blood–brain barrier, vagal nerve transport and activated leukocyte trafficking to the brain. The antidepressant effects of EVs can occur through modulation of the expression of neurotrophic factors, neurotransmitter regulation or possible supplementation of the astrocytes with glycolytic enzymes [206].

Fecal microbiota transplant (FMT) involves transferring fecal bacteria and other microbes from a healthy donor to an individual with a specific ailment. While the most established use of FMT is for *Clostridioides difficile* infection [207], there has been growing interest in its potential for treating various human diseases. Unlike probiotics, which typically consist of a limited number of bacterial strains, FMT offers the advantage of tapping into the broader bacterial diversity present in the human gastrointestinal tract, including strains not available in probiotics. Initial explorations have been made into employing FMT as a therapy for depression. In rodent experiments, FMT from healthy donors has been demonstrated to reduce depressive symptoms in animals bred for depressive tendencies or exposed to chronic stress conditions [208,209]. Moreover, FMT has yielded long-term reductions in depression symptoms (up to 3 months) in individuals with irritable bowel syndrome (IBS) [210]. Notably, FMT has been investigated as a treatment for MDD in humans, showing promise. In a groundbreaking study by Cai et al., a 79-year-old patient with severe treatment-resistant depression experienced significant improvements just two days after FMT, with her symptoms completely disappearing after six months. Concurrently, her gut microbiota composition normalized [211]. Two other MDD patients also saw improvements in depressive symptoms four weeks after FMT, with one experiencing effect lasting up to eight weeks [212]. The first randomized controlled trial of FMT for MDD reported that the intervention appeared safe and well-tolerated, enhancing patients’ quality of life, though it did not directly assess depression symptoms using verbal scales [213]. These findings suggest that FMT may represent a novel therapeutic avenue for depression by restoring the composition of the intestinal microbiota. However, its application in depression therapy, as well as other diseases, necessitates extensive and meticulous research, taking into account factors such as the microbiota profiles of patients and donors, the route and formulation of FMT, and the quantity of transplanted material. Additionally, the potential risk of serious infections linked to FMT should not be underestimated [214].

Products and dietary supplements based on microorganisms from the human gut microbiota offer the potential to alleviate depressive symptoms and associated abnormalities. They offer several advantages over traditional antidepressant drugs, being generally safe for consumption with fewer side effects. A comparative assessment of various methods for administering these preparations is provided in Table 2. However, extensive and systematic research is needed to evaluate the effectiveness of probiotics in preventing and treating depression within large human populations, as well as to establish the optimal conditions for their application.

## 6. *Faecalibacterium prausnitzii* as a Promising New-Generation Probiotic and Auxiliary Diagnostic Tool

Today, genera and species of commensal microorganisms are offered as next-generation probiotics (NGPs), identified among the intestinal microbiota when they are present in sufficient quantities, and have demonstrated promising results in terms of health promotion in model studies and have never been used in the food industry before. Such probiotics are currently under intensive study [215,216]. Among the most promising candidates are strains of the species *Faecalibacterium prausnitzii* (formerly *Fusobacterium prausnitzii*).

As mentioned above, numerous studies of the microbiota of patients with MDD revealed lower levels of *F. prausnitzii* in comparison to healthy controls [27,217]. In a preclinical study by Hao et al. on a rat model, it was shown that taking the *F. prausnitzii* strain ATCC 27766 after exposure to stress leads to an increase in SCFA levels in the cecum, an increase in the level of anti-inflammatory cytokine IL-10 in plasma, a decrease in corticosterone and IL-6 levels and suppression of increased regulation of inflammatory cytokines [186]. Also, the *F. prausnitzii* ATCC 27766 strain showed anxiolytic and antidepressant-like effects [186]. The revealed positive correlation with brain health and protective effects against neurological diseases suggested the possibility of using this strain as an additional preventive and therapeutic strategy for symptoms of depression and anxiety.

*F. prausnitzii* is one of the most common bacterial species in the colon of healthy adults, accounting for more than 5–15 percent of the total bacterial population [218,219]. Representatives of the genus *Faecalibacterium* are distributed in human populations around the world and are found in 85% of intestinal samples [220]; therefore, they are considered ubiquitous in the gastrointestinal tract of healthy people [221]. Taxonomically, *F. prausnitzii* belongs to the Firmicutes type, the Clostridia class and the Ruminococcaceae family, and currently, this species is the representative most characterized within the genus [222]. *F. prausnitzii* is a moveless Gram-positive rod that does not form spores, a strict anaerobe, extremely sensitive to oxygen [222]. The proportion of *F. prausnitzii* in the gut microbiota is flexibly affected by the colon’s physiological environment, not only the oxygen concentration but also pH and cholate [223]. A metabolic feature is the ability to produce shikimic acid, commonly found in plants, capable of protecting against inflammation caused by LPS. *F. prausnitzii* can also produce salicylic acids, which help bacteria prevent biofilm formation, which is a common feature of some infection-causing microbes [119]. *F. prausnitzii* produces important metabolites such as butyrate, which induces very low secretion of proinflammatory cytokines such as IL-12 and IFN-γ and enhanced secretion of the anti-inflammatory cytokine IL-10 in a human PBMC culture supernatant and mouse serum in a colitis model [224]. In addition, it has been demonstrated that *F. prausnitzii* supernatant reduces the intensity of inflammation by releasing metabolites that improve the functioning of the intestinal barrier and have an effect on paracellular permeability [120]. Anti-inflammatory effects were partially associated with secreted metabolites capable of blocking NF-kB activation, IL-8 production [224] and increased production of regulatory T cells [225]. In the supernatant of cultures *F. prausnitzii*, seven peptides that originate from a single microbial anti-inflammatory molecule, a 15 kDa protein MAM (ZP05614546.1), were identified [226]. *F. prausnitzii* possess a neuromodulating potential. In the study of Kovtun et al., *F. prausnitzii* entered into a signature pair with genes encoding enzymes involved in the metabolism of neurometabolites—biomarkers of depression [27].

Phylogenetic analysis of 16S rRNA sequences together with wgMLST profiles and a phylogenomic tree based on genome similarity comparisons confirmed the grouping of *F. prausnitzii* strains into different genospecies groups. Two phylogroups have been described within this species, although the actual diversity remains unknown. *F. prausnitzii* is very sensitive to changes in the intestinal environment that may limit its distribution, especially in diseased intestines. Changes in the richness and number of the population of this species have been observed in several intestinal disorders [120]. At the same time, representatives of phylogroup I of *F. prausnitzii* more often show a difference in the number and composition in the microbiota of cohorts with intestinal diseases compared to healthy subjects [227], whereas phylogroup II has limited use as a biomarker. This can be partly explained by the fact that the representation of phylogroup II is reduced to a lesser extent in intestinal diseases. It has been suggested that monitoring of *F. prausnitzii* can serve as a biomarker to assist in gut disease diagnostics.

Different studies have shown that there is actually a wide variety of genetic profiles and functions in *F. prausnitzii*. It is likely that in the coming years, there will be several species and strains associated with *F. prausnitzii,* which will improve our knowledge about these bacteria. Benevides et al. reported the existence of seven different groups in the species, based on the average values of the nucleotide identity of 16S rRNA, using 17 strains of *F. prausnitzii* [228]. Subsequently, Fitzgerald et al. updated the taxonomy of the species by analyzing the genomics of 31 strains of *F. prausnitzii* and found that the strains were divided into eight gene groups [219]. Different gene groups contain different sets of genes, which suggests that they have different properties in terms of interaction with the host [219]. This study also revealed intra-strain heterogeneity of copies of the 16S rRNA gene and emphasized that identification based on the 16S rRNA gene is not suitable for this taxon. Alternative *rpoA* gene markers have been proposed for the classification of the species, and a promising qPCR method has been developed for the separation of each group of genes. The application of the developed PCR method in six healthy adults revealed noticeable differences in the abundance and prevalence among the different targeted groups in stool samples. Further use of this developed assay will facilitate a detailed understanding of the impact of *Faecalibacterium* populations at the group level on human health and the relationship between the depletion of certain groups of *Faecalibacterium* spp. and various human diseases [229]. However, there is still not enough information about which phylogroup is important under certain conditions in the intestine. The depletion of this species is not uniform in all intestinal diseases; the use of *F. prausnitzii* as the gold standard for determining a healthy intestinal microbiota is limited. Nevertheless, these bacteria may be a good biomarker of some intestinal disorders correlated with mental illness, including depression.

Being one of the most common intestinal commensal bacteria, *F. prausnitzii* has a double effect of competitive behavior. On the one hand, it suppresses pathogenic bacteria. On the other hand, it increases the colonization of beneficial bacteria [230], maintaining a normal proportion in the GM. Thus, *F. prausnitzii*, together with other beneficial intestinal bacteria, can effectively prevent the reproduction of intestinal pathogenic bacteria such as *E. coli*, *Clostridium* and *Shigella*, while reducing the likelihood of damage to the intestinal epithelium and avoiding the activation of intestinal immune cells leading to inflammation [226]. When co-cultured with *Bacteroides thetaiotaomicron* and *Desulfovibrio piger* bacteria, *F. prausnitzii* can produce more butyric acid than they can by themselves [187,231]. This indicates that *F. prausnitzii* may rely on another gut bacterium for cross-feeding. Some studies have shown that colonization of *F. prausnitzii* requires *B. thetaiotaomicron* and *D. piger* to already exist in the intestine, which could prepare suitable conditions for the reproduction of *F. prausnitzii* by reducing the redox potential in the medium and changing the composition of nutrients [119,187].

*F. prausnitzii* isolates have common characteristics, such as lack of adhesion to epithelial cells [232], absence of plasmids and antimicrobial activity, as well as hemolytic activity and the presence of DNAse activity [233]. However, differences in enzyme production, resistance to antibiotics and immunomodulatory properties depend on the strain. Selected strains of *F. prausnitzii* with antidepressant properties may be good candidates as a next-generation probiotic recommended as an adjuvant in the treatment of depressive disorders.

It is possible to restore and maintain the number of *F. prausnitzii* not only by introducing a probiotic *F. prausnitzii* strain, but also with the help of a diet. The amount of *F. prausnitzii* increases significantly with a plant-based diet [234] and a vegan diet [235]. The addition of isoflavones and the consumption of certain types of fatty acids, such as monounsaturated fatty acids, also lead to an increase in the number of *F. prausnitzii* [236]. The use of inulin supplements by healthy volunteers for 16 days led to an increase in the intestinal populations of *Faecalibacterium* and *B. adolescentis* with a concomitant increase in butyrate production [237].

## 7. Diet for Depression Therapy

Various animal model studies indicate a direct relationship between diet, microbiota and mechanisms involved in depression [238,239]. Changes in the gut microbiota caused by diet contribute to behavioral changes that mimic the symptoms of common mental disorders such as anxiety and depression. Animal studies show that a long-term diet with a high fat content (HFD) can lead to intestinal dysbiosis with a concomitant increase in the level of peripheral cytokines and LPS, induction of TLR4 receptors and nitrosative stress, as well as a decrease in the concentration of the proteins claudin-1 and occludin in the colon [240]. Preclinical studies also show that high-calorie diets lead to a decrease in cognitive flexibility, as well as a violation of social perception and object recognition [241,242]. Human studies show that a Western diet with a proinflammatory function, consisting of a high percentage of fats and sugars, and excessive alcohol consumption correlate with MDD and impaired intestinal barrier function [243,244]. In contrast, beneficial nutrients, such as fiber, polyunsaturated fatty acids and polyphenols, positively affect brain health through the direct involvement of the microbiota, including in ensuring the bioavailability of these compounds [245,246]. The Mediterranean diet or other anti-inflammatory diets may become a new strategy to counteract the inflammatory condition associated with the occurrence and severity of mental disorders [247,248]. Omega-3 fatty acids eicosapentaenoic acid and docosahexaenoic acid, polyunsaturated fatty acids, which are found in high concentrations in marine foods such as salmon, have anti-inflammatory properties and can improve clinical outcomes [249]. There is a prospect that a diet that positively affects the microbial composition and permeability of the intestine and BBB can affect humoral and immune mechanisms, including glial functions, with subsequent effects on mental and physical health.

Due to the high load of oxidative stress reported in people with mental disorders [250], improving the quality of the diet may be a viable intervention to replenish depleted antioxidant defenses. A diet high in nutrients with antioxidant properties leads to a decrease in markers of oxidative stress, such as F2-isoprostanes and oxidized low-density plasma lipoprotein [251]. Studies reveal that depression is associated with lower intake of antioxidants such as vitamins A, C and E; selenium; zinc; and B vitamins (B6, folate, and B12) [55].

Fermented foods containing prebiotics and biogenics are another group of foods potentially capable of participating in the connection between the intestine and the brain [252]. Some studies have shown promising results in improving mood after eating fermented foods [252]. Due to the non-viability and variable colonizing ability of probiotics, a diet that includes a wide range of plant food sources for bacteria may be preferable for stimulating the growth of probiotic strains.

Thus, a healthy diet during depression therapy, along with the administration of probiotics and psychobiotics, can potentially improve the course of the disease and contribute to the progress of treatment.

## 8. Conclusions

It is now fairly well known that depressive disorders, like other mental diseases, are caused and/or accompanied by neuroinflammatory processes. The gut microbiome is an integrator and target for the impact of various physical–chemical, social, ecological and other stress factors, as well as products to support its vital functions. By conducting multichannel communication with the host organism and supporting its positive homeostasis, the gut microbiome acquires a key role as an indicator of human health. Naturally, the following question arises: what parameters (biomarkers) characterize the state of the microbiome in the norm? Such a parameter could be a metagenomic signature—a matrix describing which genes and in what quantity are contained in the microbiota. The matrix can be considered at various taxonomic levels (genus, species, phylogroup or strains) and different key groups of genes involved in the functioning of this or that system of the human body. Thus, certain signatures of the gut microbiome can characterize its neuromodulatory, immunomodulatory and antioxidant potential. The totality of these potentials of the gut microbiome characterizes a human’s ability to resist stresses of various etiologies and the impact of other adverse environmental factors. Therefore, at the current level of microbiome research, metagenomic signatures are adequate biomarkers of the microbiome’s condition. In this review, we have tried to analyze the data available today on biomarker taxa and metabolites of gut microbiota that can be considered as metagenomic signatures of depressive disorder.

There is relatively little research in the field of the development of a new generation of probiotics and LBPs for which the mechanism of action has been established and pharmacologically active ingredients determining antidepressant properties have been identified. There is a lack of a technological chain for searching for and promoting candidates for drugs from the gut microbiome of a certain category of people and for the identification of combinations of given genes with neuromodulatory, immunomodulatory and anti-inflammatory activity in the genomes of selected strains. The selection and subsequent screening of strains most relevant to and adequate for specific depressive disorders, including depression caused by extreme conditions, chronic social depression and depression associated with post-COVID syndrome, are required. The mandatory testing of drug candidates focusing on restoring the signature characteristic of a healthy person is also needed. The difficulty in using a combination of bacterial strains of different species lies in the lack of knowledge about their individual and synergistic effects or incompatibility when acting on the organism. The use of fecal transplantation technology, the so-called cocktail of strains and new-generation probiotics have certain success and carry threats to the safety of their use. Some of them can be avoided by using characterized postbiotic drugs obtained on their basis. For this, it is necessary to use metabolomic, proteomic and other approaches to identify and characterize biologically active substances in their composition and the absence of substances toxic to humans.

The effect of existing antidepressants on the microbiota has not been sufficiently studied. However, knowledge in this area will help improve existing treatment. Also, when developing new drugs, their modifying effect on the gut microbiome should be taken into account. Thus, this topic has great advantages for both diagnosis and therapy. Further research in this area will help create an entirely new class of antidepressants aimed at modifying the gut microbiota. For the creation of bacterial drugs aimed at restoring the microbiome and the prevention and combined treatment of depressive and other psychiatric disorders, it is necessary today to use revolutionary technologies and conceptual breakthroughs in the field of interdisciplinary research of the human gut microbiome, including metagenomic and omics technologies and artificial intelligence technologies (machine learning, neural networks), to integrate analyses of the microbiome and search for products with given properties. An understanding of the epigenetic mechanisms of regulation and restoration of human health through targeted correction of the microbiome using LBPs, postbiotics and corresponding ingredients derived from them in food products is also required.

Considering the certain region-specificity of the composition of the gut microbiome, it seems appropriate to start forming a number of large national and international projects studying “microbiome-directed products for the correction of mental diseases”.

## Figures and Tables

**Table 1:** The effects of probiotics on patients with depression in double-blind, placebo-controlled trials.

NN	Probiotics, Prebiotics and Antidepressants Used; Daily Dose (CFU)	Duration of the Study, Weeks	Participant Type	Study Cohort Size	Changes in People’s Behavior	Physiological Changes	References
1	*L. acidophilus*, *L. casei*, *B. bifidum* 2 × 10^9^ CFU/g each	8	Patients with clinically recognized MDD	40 (20 probiotic group, 20 placebo group)	↓ BDI	↓ serum insulin levels ↓ homeostasis model of assessment of insulin resistance (HOMA-IR) ↓ serum hs-CRP concentrations ↑ plasma total glutathione levels	[169]
2	*L. helveticus* R0052 *B. longum* R0175 ≥3 × 10^9^	8	Human volunteers with symptoms of depression	69 (33 probiotic group, 36 placebo group)	No significant group differences in MADRS; QIDS-SR16; DASS-42	No significant difference between groups on any blood-based biomarker	[157]
3	*B. longum* NCC3001 10^10^ CFU	6	IBS patients with mild to moderate depression and/or anxiety	44 (22 probiotic group, 22 placebo group)	↓ HADS depression ↑ Life quality No effect on anxiety	↓ Responses to negative emotional stimuli in the amygdala and fronto-limbic regions ↓Urine levels of methylamines and aromatic amino acids metabolites No effect on fecal microbiota profiles, serum inflammatory markers, BDNF, substance P and 5-HT levels	[143]
4	Familact H^®^ donated by Zist Takhmir Co., Tehran, Iran: *L. casei* *L. acidophilus*, *L. bularigus*, *L. rhamnosus*, *B. breve*, *B. longum*, *S. thermophilus* 3 × 10^7^–2 × 10^8^ CFU each; Prebiotic fructooligosaccharide; fluoxetine 20 mg	10	Patients with clinically recognized moderate depression	40 (20 synbiotic group, 20 placebo group)	↓ HDRS	-	[145]
5	*L. helveticus* R0052, *B. longum* R0175 10^10^ CFU (probiotic group); galactooligosaccharide (prebiotic group); SSRI (sertraline, fluoxetine, citalopram) or tricyclic amitriptyline antidepressants (all three groups)	8	Patients with clinically recognized mild to moderate MDD	81 (28 probiotic group, 27 prebiotic group, 26 placebo group)	↓ BDI depression in probiotic group compared to the placebo and prebiotic group	↓ Kynurenine/tryptophan in plasma in the probiotic group compared to the placebo group ↑ Tryptophan/isoleucine in the probiotic group compared to the placebo group	[158]
6	*Clostridium butyricum* MIYAIRI 588 60 mg/day antidepressants	8	Patients with treatment-resistant MDD	40 (20 probiotic group, 20 placebo group)	↓ HAMD ↓ BDI ↓ BAI	-	[170]
7	*L. plantarum* 299v 2 × 10^10^ CFU antidepressants	8	Patients with MDD	60 (30 probiotic group, 30 placebo group)	↑ APT ↑ CVLT No statistical significance	↑ 3HKYN:KYN ↓ KYN No statistical significance	[171]
8	*Bacillus* *coagulans* MTCC 5856 2 × 10^9^ CFU	13	IBS patients with MDD	40 (20 probiotic group, 20 placebo group).	↓ HAM-D ↓ MADRS ↓ CGI-I ↓ CGI-S ↓ Dementia—TFS ↓ mESS	↓ Serum myloperoxidase	[152]
9	Bac-Set Forte (Probiotics International Ltd., Somerset, UK): 14 strains *L. casei*, *L. plantarum*, *L. rhamnosus*, *B. bifidum*, *B. breve*, *B. longum*, *L. acidophilus*, *L. lactis ssp. lactis*, *S. thermophiles*, *B. infantis*, *L. delbrueckii ssp. bulgaricus*, *L. helveticus*, *L. salivarius*, *L. fermentum* 6 × 10^9^ CFU Cipralex 10 mg	8	Patients diagnosed with mild or moderate depressive episodes	119 (60 probiotic group, 59 placebo group)	↓ HDRS-17	↓ cortisol, IL-6, TNF-alfa in blood serum; NO, dopamine in blood plasma	[172]
10	*B. breve* CCFM1025 10^10^ CFU	4	Patients diagnosed with MDD	45 (20 probiotic, 25 placebo group)	↑ HDRS-24 ↑ MADRS	↑ Serum serotonin turnover	[151]
11	Vivomixx/Visbiome: 8 strains: *S. thermophilus*, *B. breve*, *B. longum*, *B. infantis*, *L. acidophilus*, *L. plantarum*, *L. paracasei*, *L. helveticus* 9 × 10^11^ CFU Plus treatment as usual	4	Patients with current depressive episodes	45 (21 probiotic group, 26 placebo group)	↓ HAM-D	Probiotics maintained alfa microbial diversity in the gut, ↑ the abundance of the genus *Lactobacillus*	[177]
12	Vivomixx/Visbiome: 8 strains: *S. thermophilus*, *B. breve*, *B. longum*, *B. infantis*, *L. acidophilus*, *L. plantarum*, *L. paracasei*, *L. helveticus* 9 × 10^11^ CFU Plus treatment as usual	4	Patients with current depressive episodes	32 (18 placebo group, 14 probiotics group)	See N 11	A multimodal neuroimaging approach Probiotics induced structural and functional changes in the brain correlating with reduction in depressive symptoms	[178]
13	Vivomixx/Visbiome: 8 strains: *S. thermophilus*, *B. breve*, *B. longum*, *B. infantis*, *L. acidophilus*, *L. plantarum*, *L. paracasei*, *L. helveticus* 9 × 10^11^ CFU Plus treatment as usual	4	Patients with current depressive episodes	43 (19 probiotic group, 24 placebo group)	↑ VLMT See N 11	Remediated hippocampus function in the probiotic group BDNF serum level—no significant difference between groups	[179]
14	Bio-Kult^®^ Advanced, ADM Protexin Ltd, Somerset, UK., 14 species: *B. subtilis*, *B. bifidum*, *B. breve*, *B. infantis*, *B. longum*, *L. acidophilus*, *L. delbrueckii* ssp. *bulgaricus*, *L. casei*, *L.s plantarum*, *L. rhamnosus*, *L. helveticus*, *L. salivarius*, *L. lactis* ssp. *lactis*, *S. thermophilus* 2× 10^9^ CFU	4	Volunteers with moderate depression	71 (36 probiotic group, 35 placebo group)	↓ PHQ-9, but these did not correlate with the changes in emotional processing	Probiotic did not alter salivary cortisol or circulating CRP concentrations	[144]

Abbreviations: Attention and Perceptivity Test (APT), Beck Anxiety Inventory (BAI), Beck Depression Inventory (BDI), California Verbal Learning Test (CVLT), Center for Epidemiological Studies Depression Scale (CES-D), Clinical Global Impression Severity Rating Scale (CGI-S), Clinical Global Impression-Improvement rating (CGI-I), Dementia—total frequency scoring (Dementia—TFS), Depression, Anxiety and Stress Scale (DASS-42), Hamilton Depression Rating Scale (HDRS, HAM-D), Hospital Anxiety and Depression Scale (HADS), Modified Epworth Sleepiness Scale (mESS), Montgomery–Åsberg Depression Rating Scale (MADRS), Outcome Questionnaire 45 (OQ45), quality of life (QoL), Patient Health Questionnaire-9 (PHQ-9), Quick Inventory of Depressive Symptoms (QIDS-SR16), Verbal Learning Memory Test (VLMT).

**Table 2:** Comparing different approaches to utilizing microorganisms as antidepressants.

Type of Product	Optimal Methods of Administration	State of Research	Advantages	Disadvantages
Probiotic	Capsules; substantial doses (10^9^–10^10^ CFU); prolonged usage (8 weeks); utilization of multi-strain probiotics; utilization as symbiotics; use in conjunction with antidepressants; treatment for clinical depression.	Thoroughly investigated in preclinical and clinical studies.	Specific strains exhibit activity reliably and are relatively easy to obtain.	They are living organisms, which can induce inflammation and participate in the transfer of antibiotic resistance genes.
Prebiotic	Administration as symbiotic.	Isolated studies conducted on animals and humans.	They are not living organisms and do not pose corresponding risks.	When used as monopreparations, they seldom exhibit activity.
Postbiotic	Differ for different postbiotics.	Studied in isolated preclinical and clinical trials, primarily utilizing inactivated bacterial cells.	They are not living organisms and do not carry associated risks; relatively easy to standardize.	Often involves more complex and costly manufacturing processes.
FMT	Selection of a suitable and reliable donor; other conditions are not yet developed.	Individual preparations were investigated in animals and have been used in several instances in humans.	Rapid and significant normalization of the composition of the gut microbiota; microbiota changes can persist for an extended period.	Obtaining them is complex; they are not amenable to standardization (each preparation is unique); they are living organisms and can pose a threat to patient health.
